# Orally Disintegrating Tablets Containing Melt Extruded Amorphous Solid Dispersion of Tacrolimus for Dissolution Enhancement

**DOI:** 10.3390/pharmaceutics10010035

**Published:** 2018-03-16

**Authors:** Poovizhi Ponnammal, Parijat Kanaujia, Yin Yani, Wai Kiong Ng, Reginald B. H. Tan

**Affiliations:** 1 Institute of Chemical and Engineering Sciences, 1, Pesek Road Jurong Island, Singapore 627833, Singapore; poovizhi1387@gmail.com (P.P.); yinyani@gmail.com (Y.Y.); ng_wai_kiong@ices.a-star.edu.sg (W.K.N.); 2 Department of Chemical and Biomolecular Engineering, National University of Singapore, Singapore 117585, Singpore; 3 Department of Pharmacy, National University of Singapore, Singapore 117559, Singpore

**Keywords:** melt extrusion, amorphous solid dispersion, dissolution enhancement, tacrolimus, orally-disintegrating tablets

## Abstract

In order to improve the aqueous solubility and dissolution of Tacrolimus (TAC), amorphous solid dispersions of TAC were prepared by hot melt extrusion with three hydrophilic polymers, Polyvinylpyrrolidone vinyl acetate (PVP VA64), Soluplus^®^ and Hydroxypropyl Cellulose (HPC), at a drug loading of 10% *w*/*w*. Molecular modeling was used to determine the miscibility of the drug with the carrier polymers by calculating the Hansen Solubility Parameters. Powder X-ray diffraction and differential scanning calorimetry (DSC) studies of powdered solid dispersions revealed the conversion of crystalline TAC to amorphous form. Fourier transform Infrared (FTIR) spectroscopy results indicated formation of hydrogen bond between TAC and polymers leading to stabilization of TAC in amorphous form. The extrudates were found to be stable under accelerated storage conditions for 3 months with no re-crystallization, indicating that hot melt extrusion is suitable for producing stable amorphous solid dispersions of TAC in PVP VA64, Soluplus^®^ and HPC. Stable solid dispersions of amorphous TAC exhibited higher dissolution rate, with the solid dispersions releasing more than 80% drug in 15 min compared to the crystalline drug giving 5% drug release in two hours. These stable solid dispersions were incorporated into orally-disintegrating tablets in which the solid dispersion retained its solubility, dissolution and stability advantage.

## 1. Introduction

Tacrolimus (TAC) is a potent immunosuppressive drug widely used to prevent organ rejection of liver and kidney transplants and less frequently in heart, lung and heart lung transplants. TAC acts by engaging an immunophilin, FK506-binding protein-12 (FKBP12) and forming a complex which inhibits calcineurin with much more potency than cyclosporine [1,2]. TAC is a highly hydrophobic drug with aqueous solubility of 0.7–2 µg/mL [3,4]. It falls under class 2 of the Biopharmaceutics Classification System (BCS) for drugs and permeability of TAC has been reported to be approximately 1.4 × 10^−4^ cm/s [5,6]. TAC is marketed as a capsule (Prograf^®^) containing solid dispersion of drug with hydroxypropyl methylcellulose in order to improve dissolution and bioavailability [4,7] and extended release tablet (Envarsus XR^®^) prepared by melt agglomeration technology [8]. It exhibits low bioavailability (10–25%) and significantly variable pharmacokinetics due to its poor aqueous solubility, extensive metabolism by intestinal and hepatic cytochrome P450 3A4 enzyme [9] along with the effects of P glycoprotein efflux in the intestine [5,10,11]. TAC has a narrow therapeutic window (5–20 ng/mL) and overexposure increases the risk of nephrotoxicity and neurotoxicity [12,13] whereas low level can lead to graft rejection.

Several attempts have been made to improve the dissolution rate of TAC using solid dispersion formulation with various hydrophilic excipients. Solid dispersions of TAC with poloxamer 407, polyvinyl alcohol and sodium dodecyl sulfate were prepared using ultra-rapid freeze-drying. Upon dissolution in acidic media, these solid dispersions formed supersaturated solution and the solid dispersion with poloxamer 407 showed 1.5 fold improvement in oral bioavailability [14]. Effect of the formulation method on the dissolution rate was studied by preparing solid dispersions by spray drying with solvent evaporation/solvent wetting/surface attach method with water. The formulations prepared by solvent evaporation method produced amorphous dispersion which showed a 15 fold increase in dissolution [3]. TAC was formulated as solid dispersion with amino alkyl methacrylate copolymer (Eudragit E^®^) with hydrochloric acid. The solubility and dissolution of the drug was improved several fold with no re-precipitation of drug for up to 24 h [15,16]. Solubility and dissolution enhancing effect of different types of cyclodextrins was studied by complexing TAC with cyclodextrins [17]. Proliposomes for TAC were formulated using various lipids by the thin film hydration method. In Vitro studies show that the drug release from the optimized proliposome formulation was significantly higher than that of the pure drug, this has also been correlated to in vivo studies which showed promising results [18].

These formulation approaches were based on increasing dissolution rate of TAC and lack in preventing or reducing the effect of intestinal enzymatic degradation and p-glycoprotein efflux pump on bioavailability of TAC. An inhalable formulation of TAC was developed by spray drying TAC with lung lipids like DPPC and DPPG at 100 °C in a molar ration of 3:1 to mimic the lung surfactant layer [19]. In a clinical study, 17 heart and lung transplant patients were given TAC sublingually twice a day by administering contents of marketed capsuled below the tongue for 15 min and patients were advised to not to swallow the saliva during this 15 min period. The sublingual delivery of TAC was able to generate therapeutic levels of TAC in blood without initial spike in blood levels. The sublingual TAC administration was found to be effective in replacing intravenous injection [20]. A fast disintegrating tablet containing solid dispersion of TAC was prepared and evaluated for dissolution and stability. The solid dispersion was prepared by solvent removal method with three different stabilizers namely inulin 1.8 kDa, inulin 4 kDa and PVP K 30. The tablet prepared from solid dispersion containing 10% of TAC showed optimal results and inulin 1.8 kDa tablet showed excellent stability and retention of fast dissolution property [21].

In this research work, an attempt was made to stabilize TAC in amorphous form by preparing solid dispersions with different polymeric excipients using melt extrusion and incorporating into orally-disintegrating tablets (ODTs). Melt extrusion is a continuous and industrially feasible process which does not involve the use of solvents. It is easily scalable and has few processing steps [22]. This work shows the development of formulations of TAC, their characterization and a study of their dissolution profiles and storage stability. The advantages of the amorphous state, the method of production and the dosage form have been harnessed to produce formulations by synergizing the benefits.

## 2. Materials and Methods 

### 2.1. Materials

TAC was purchased from Concord Biotech Ltd., Ahmedabad, India. PVP VA64 (Kollidon VA 64) and Soluplus^®^ were kindly gifted by BASF, Ludwigshafen, Germany. Hydroxypropyl Cellulose (HPC, LF grade) was received as sample from Ashland (Ashland Inc. Covington, KY, USA) Micro Crystalline cellulose (Avicel PH101), Mannitol, Magnesium stearate and other chemicals used were purchased from Sigma Aldrich (Sigma, St. Louis, MO, USA). HPLC grade methanol and acetonitrile were purchased from Fischer Scientific (Fischer Scientific Pte. Ltd, Pandan Cres., Singapore).

### 2.2. Hansen Solubility Parameter and Excipient Selection 

**Figure 1:**
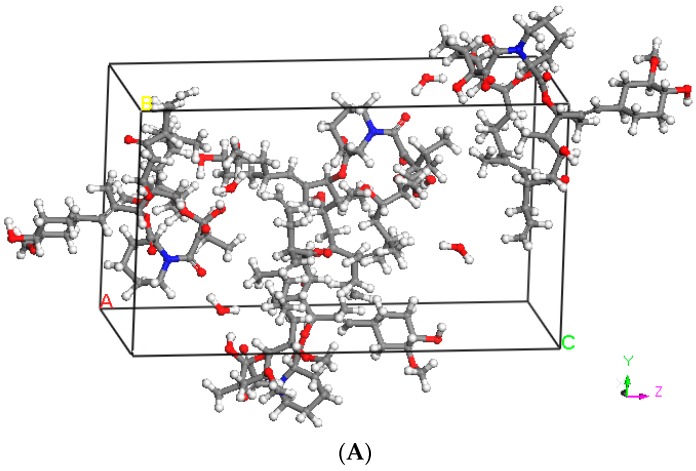

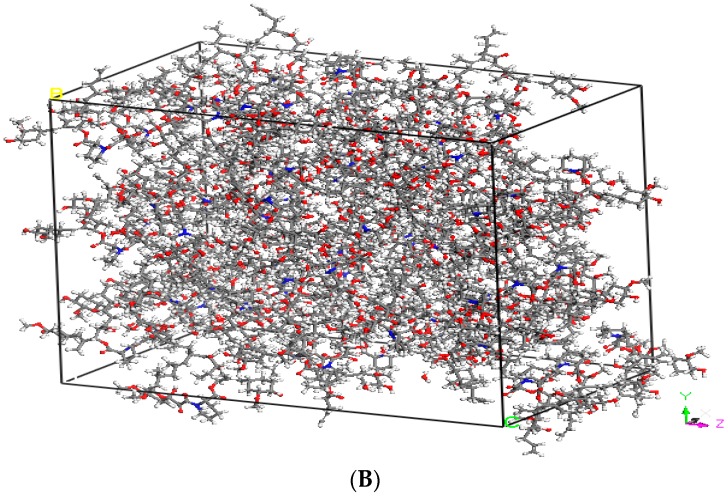
(**A**) Crystal structure of tacrolimus (TAC) (**B**) equilibrated amorphous cell of TAC (72 molecules).

Molecular dynamics (MD) simulations were carried out using the Materials Studio Version 7.0.100 (Accelrys Software Inc, San Diego, CA, USA) for TAC [23]. The crystal structure (Figure 1A) was obtained from the Cambridge Structural Database, Ver. 5.26 (Cambridge Crystallographic Data Centre Cambridge, UK). The space group of the crystal is P212121 (orthorhombic with the unit cell dimensions of a = 10.939 Å, b = 15.878 Å, c = 27.184 Å, and Z = 4). The crystal structure was extended to 3 × 3 × 2 unit cells (72 TAC molecules). The COMPASS [24] (condensed-phase optimized molecular potentials for the atomistic simulation studies) force field was used to model the atomic interactions for TAC molecules. COMPASS force field model gives densities of 1.104 g/cm^3^ for pure TAC. The integration time step used was 1 fs. Ewald summation was used to enable the long-range interactions. A cutoff radius of 12.5 Å was used for both non-bonded and electrostatic interactions. Simulation in the NPT (constant number of particle, constant pressure, and constant temperature) ensemble was conducted at 298 K for 2 ns to ensure that TAC system reaches equilibration condition. The equilibrated amorphous cell of TAC is shown in Figure 1B. Equilibration was determined by observing the change in the thermodynamic properties (energies, temperatures, and densities) as a function of time. The system was concluded to have reached equilibration condition if these properties showed sufficiently small variations over time. The required time was less than 100 ps. The Nose/Hoover thermostat [25] and Berendsen barostat [26] were used to control the temperature and pressure, respectively. The production run was done by choosing three different trajectories (at time step of 1 ns, 1.5 ns, and 2 ns) from the equilibrated system. Energy minimization was then performed for the three sets of data, followed by MD simulation in NVT (isothermal) ensemble at 298 K for 100 ps. The final 50 ps were used to calculate the Hansen solubility parameter. The three data sets were then averaged to obtain the solubility parameter for TAC. The solubility parameters of the polymers were taken from literature.

### 2.3. Thermogravimetric Analysis

In order to study the thermal stability of TAC alone and in combination with polymers, thermogravimetric analysis (TGA) was performed using a TGA Q500 V6.7 build 203 (TA Instruments, New Castle, DE, USA). Alumina crucibles were used and the sample weight ranged from 5–15 mg. The sample was heated from 25 to 200 °C at the rate of 10 °C/min and weight loss was recorded.

### 2.4. Differential Scanning Calorimetry

A hyper differential scanning calorimetry (DSC) (PerkinElmer instruments, Shelton, CT, USA) was used to record the thermogram. The enthalpic response was calibrated with Indium and zinc. An empty sealed pan was used as reference. The samples were kept under isothermal condition at 25 °C for 10 min in sealed standard aluminum pans. DSC study was performed at 10 °C min^−1^ from 25 to 160 °C under a nitrogen flow rate of 20 mL min^−1^. A second reheating step at 10 °C/min to 160 °C after cooling to −50 °C was added to DSC of pure TAC to determine the glass transition temperature (T_g_). The data treatment and integration were done by Pyris Analysis (PerkinElmer, instruments, Shelton, CT, USA).

### 2.5. Preparation of Solid Dispersions by Hot Melt Extrusion (HME)

The physical mixtures were prepared by geometric mixing of the individually weighed polymers and drug. The physical mixture was placed in a powder mixer (Alphie powder mixer, Hexagon product development, Vadodara, Gujarat, India) for 20 min to ensure homogeneity. A preheated co-rotating twin screw melt extruder (Prism Eurolab 16 Melt extruder from Thermo Scientific, Karlsruhe, Germany) having a horizontally split barrel with length to diameter ratio of 25:1 and 15.6 mm diameter screws was used for the extrusion. About 30–40 g of the physical mixture containing 10% *w*/*w* of TAC was manually fed to preheated twin-screw extruder rotating at a speed of 200 rpm. In order to reduce the residence time in the heated barrel, the physical mixture was fed from zone 3. The temperatures used in the different zones are shown below.
Barrel Zone123456Rod dieTemperature (°C)not usednot used70125135140135

A rod die with a 2 mm orifice was used for the extrusion of solid dispersion in the shape of cylindrical strands. A small (40 cm) conveyor belt was used to collect and air-cool the extrudates. Extrudes were stored in screw-capped glass bottles at room temperature (25 °C) under low humidity (25% relative humidity).

The extrudates were milled (MM 200 Retsch GmbH, Haan, Germany) for 2 min at a frequency of 30 Hz in a stainless-steel ball mill using a 1.5-cm diameter stainless steel ball. The powdered solid dispersions were sieved and 50–250 µm fraction was collected and stored in screw-capped glass bottles in dry cabinet (25% RH) at room temperature. The sieved fraction of solid dispersion was used for the characterization and analysis.

### 2.6. High Performance Liquid Chromatography (HPLC) Analysis

The analysis of the drug was done using an Agilent HPLC (Agilent Technologies, Santa Clara, CA, USA). A Zorbax Eclipse Plus C18 column was used with an isocratic elution method where the mobile phase was a mixture containing 45% Methanol, 45% Acetonitrile and 10% Water at a flow rate of 1.5 mL/minute and injection volume of 10 µL. The column was maintained at a temperature of 40 °C while the sample holder was kept at 4 °C and the UV detector was set to 214 nm. The samples for drug estimation were prepared in methanol and TAC was eluted at a retention time of 3.3 ± 0.1 min. For samples from dissolution experiments, the samples were directly injected into the column after filtration and analyzed. Calibration was performed in both methanol and dissolution medium between 0 to 100 mg/L [27].

### 2.7. Microscopy and Imaging

An Olympus polarization microscope BX51 (Olympus Corporation, Tokyo, Japan) was used along with a Sony digital color video camera (Sony Corporation, Tokyo, Japan) to capture images using the analySIS pro software (Soft Imaging Systems, Olympus Corporation, Tokyo, Japan). The extruded strands and milled powders were observed under a microscope under both polarized and ordinary light.

### 2.8. Powder X-ray Diffraction

The powder X-ray diffraction analysis was done on a Bruker D8 (Bruker AXS GmbH, Karlsruhe, Germany) Advance X-ray diffractometer using Cu Kα radiation (α = 1.5406 Å) with a scanning rate of 1°/min and scanning angles between 4° to 50°.

### 2.9. Fourier Transform InfraRed (FTIR) Spectrophotometric Analysis

FTIR was performed on a BioRad Spectrophotometer (Bio Rad, Philadelphia, PA, USA). The powder samples were made into KBr pellets and the transmittance in the wave number range of 4000 to 400 cm^−1^ was measured for 64 scans.

### 2.10. Dissolution Testing

Dissolution studies were done in USP II paddle dissolution apparatus (Agilent 708-DS, Agilent Technologies, Santa Clara, CA, USA). 250 mL of 0.1% sodium lauryl sulfate (SLS) in de-ionized water was used as the medium of dissolution [28]. Crystalline TAC or powdered solid dispersion equivalent to 5 mg TAC was added to the dissolution tank. The temperature of the water bath was set at 37 ± 0.5 °C and the paddle speed was set at 100 rpm. 1 mL of sample was withdrawn at each time point, syringe filtered and analyzed by High Performance Liquid Chromatography (HPLC) as described above.

### 2.11. Amorphous Stability Testing

The solid dispersion samples were stored in capped glass vials under accelerated conditions in a stability chamber (Climacell EVO, MMM Group, München, Germany) set at 40 °C and 75% RH. The storage stability testing was done for 3 months. The solid dispersion stability in terms of drug content, amorphous nature and retention of dissolution enhancement was tested.

### 2.12. Orally-Disintegrating Tablet (ODT) Compression and Characterization

The solid dispersion powders were incorporated into ODTs. The physical mixtures of the excipients were compressed using a hand operated Carver tablet press and a flat faced tablet punch (10 mm diameter). Two hundred and fifty mg of physical mixture containing the excipients and the solid dispersion was weighed into the die and the tablet was compressed by applying a force of 1 ton for 10 s. The Pharmatron Dr. Schleuniger Multi test 50 (SOTAX AG, Aesch, Switzerland) was used for hardness testing and it gave the hardness value in kilopond (kP). The prepared tablets were also evaluated for friability by Agilent Technologies 250 Friabilator and disintegration time by Agilent Technologies 100 Automated Disintegration (Agilent Technologies, Santa Clara, CA, USA).

## 3. Results

### 3.1. Hansen Solubility Parameter and Excipient Selection

The solid-solid miscibility of drug and polymers can be predicted by calculating Hansen solubility parameters (*δt*) from the chemical structure. The solubility parameter of TAC was calculated using molecular dynamic simulation using the method reported by Gupta and coworkers [29]. The difference between the solubility parameters (Δ*δt*) of two materials is known as interaction parameter and is indicative of likely miscibility or immiscibility. Compounds with Δ*δt* value 7.0 MPa^1/2^ or less are most likely to be miscible whereas compounds with value greater than 10.0 MPa^1/2^ are most likely immiscible [30]. The interaction parameter (Δ*δt*) for TAC and all polymers is less than 1, so it is most likely that drug and polymer melt would be miscible during melt extrusion. The carrier polymers were selected based on their glass transition temperatures and the Hansen solubility parameters (Table 1).

### 3.2. Thermal Stability of Tacrolimus (TAC)

Hot melt extrusion involves the use of temperature and shear for processing physical mixtures into solid dispersions. The thermal stability of materials (both drug and excipients) will play a crucial role in the selection of extrusion parameters.

**Figure 2:**
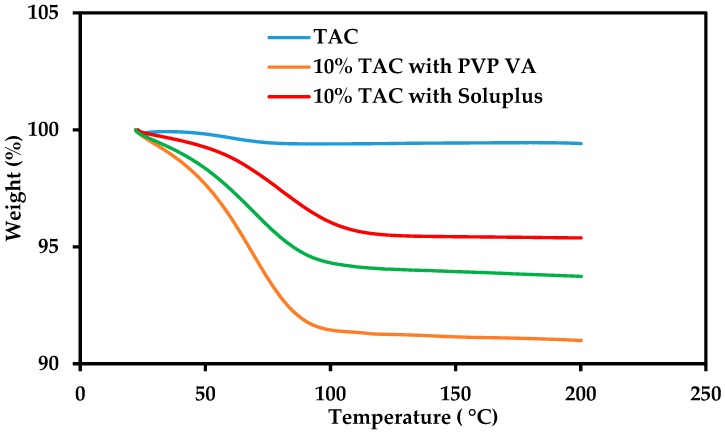
TGA of crystalline TAC and physical mixtures with PVP-VA 64, Soluplus and HPC containing 10% *w*/*w* TAC.

The TGA of pure crystalline drug shows that it begins decomposing beyond 200 °C. The physical mixtures of TAC with polymers showed a 5–10% weight loss at 100 °C which could be attributed to the evaporation of moisture present in the polymers (Figure 2). The DSC of TAC showed a melting peak at 132.8 °C during the first heating cycle. After cooling at 50 °C/min to −50 °C, and reheating at 10 °C/min, the melting endotherm of the drug disappeared and glass transition temperature (T_g_) was observed at 78.8 °C confirming conversion of crystalline TAC to amorphous form by melting and rapid cooling in the DSC. These results show that TAC can be converted to its amorphous form by melting and rapid cooling [21,33].

### 3.3. Hot Melt Extrusion 

**Figure 3:**
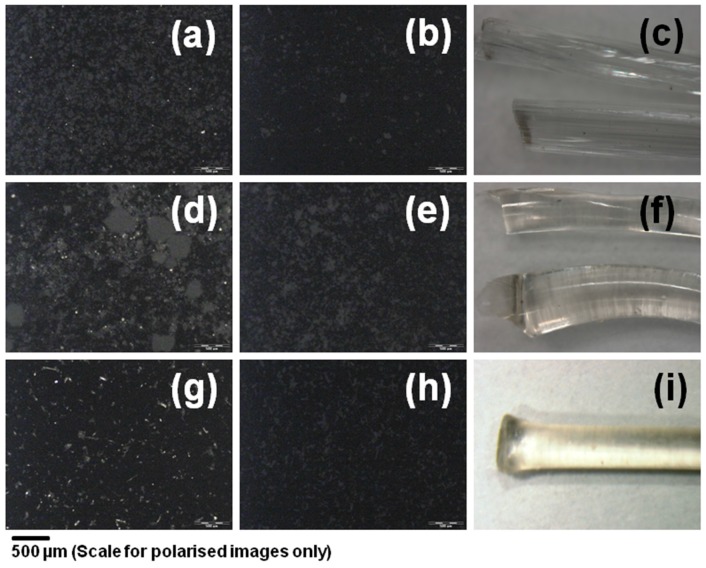
Polarized light photomicrographs of (**a**) 10% TAC PVP-VA 64 physical mixture, (**b**) 10% TAC PVP-VA 64 extruded and milled powder, (**d**) 10% TAC Soluplus physical mixture (**e**) 10% TAC Soluplus extruded and milled powder (**g**) 10% TAC HPC physical mixture, (**h**) 10% TAC HPC extruded and milled powder, and optical photomicrograph of Extruded strands of (**c**) PVP VA 64 (**f**) Soluplus and (**i**) HPC containing 10% *w*/*w* TAC.

The extruded solid dispersions are clear, transparent and brittle strands when observed under a stereo microscope equipped with a digital camera (Leica MZ 16, Leica, Wetzlar, Germany) (Figure 3c,f,i). The milled powder observed under polarized light showed that there were no crystalline particles present in the extruded formulation (Figure 3b,e,h), whereas the corresponding physical mixture showed presence of crystalline TAC (Figure 3a,d,g). TAC content in the extruded and milled solid dispersions was determined by HPLC method. The drug loading efficiency was estimated to be 102 ± 3%, 88 ± 1% and 79 ± 8% of the expected drug content (10% *w*/*w*) in PVP VA 64, Soluplus and HPC solid dispersions respectively. The reduction of TAC content in case of Soluplus and HPC could be due to degradation of TAC during extrusion.

### 3.4. Differential Scanning Calorimetry (DSC) Analysis

**Figure 4:**
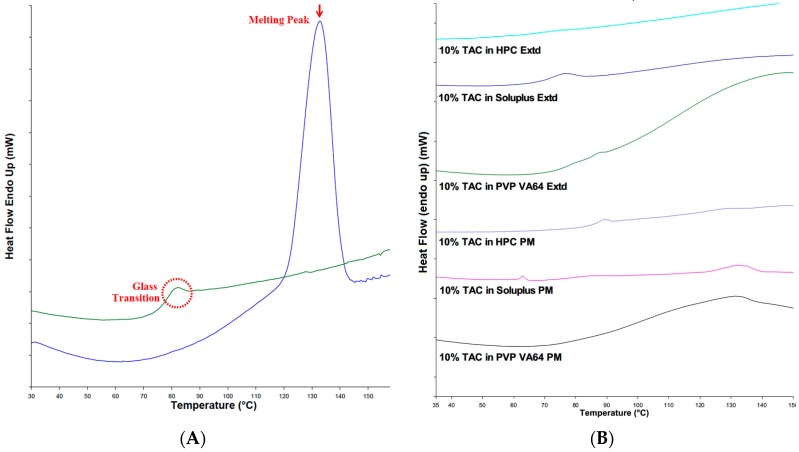
(**A**) DSC thermograms of TAC, first heating cycle showing melting peak and second heating cycle showing glass transition; (**B**) DSC of physical mixtures and formulations containing TAC.

The DSC thermograms of the physical mixtures of drug and polymer showed a melting peak at 132 °C (see Figure 4A) corresponding to the melting peak of the pure drug while the extruded solid dispersions did not have a melting peak of TAC indicating the absence of crystalline TAC in the solid dispersion. This could be attributed to the conversion of crystalline TAC to amorphous form (Figure 4B).

### 3.5. Powder X-ray Diffraction

**Figure 5:**
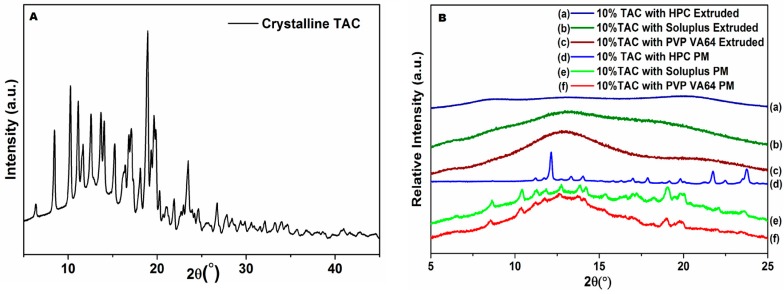
X-ray diffraction patterns of (**A**) TAC and (**B**) physical mixtures and solid dispersion containing 10% TAC.

The diffraction pattern of TAC (Figure 5A) shows characteristic peaks at 2θ values of 8.5, 10.1, 11.2, 19 and 23.5 [4,15]. The powder X-ray diffraction (PXRD) analysis of the physical mixtures shows peaks corresponding to the pure drug while the extruded solid dispersion powders exhibited an amorphous halo (Figure 5B) with all three polymers. These, along with the DSC results confirm that TAC is present in the amorphous form in the extruded samples.

### 3.6. FTIR

The FTIR spectra of TAC showed absorption bands of C-O-C (ether) stretching vibrations at 1173 and 1090 cm^−1^, C-O (ester) stretching vibration at 1194 cm^−1^, C=O (keto-amide) and C=C stretching vibration at 1640 cm^−1^, C=O (ester and ketone) stretching vibration peak at 1740, 1724 and 1694 cm^−1^, and O-H stretching vibration at 3440 cm^−1^ [34].

**Figure 6:**
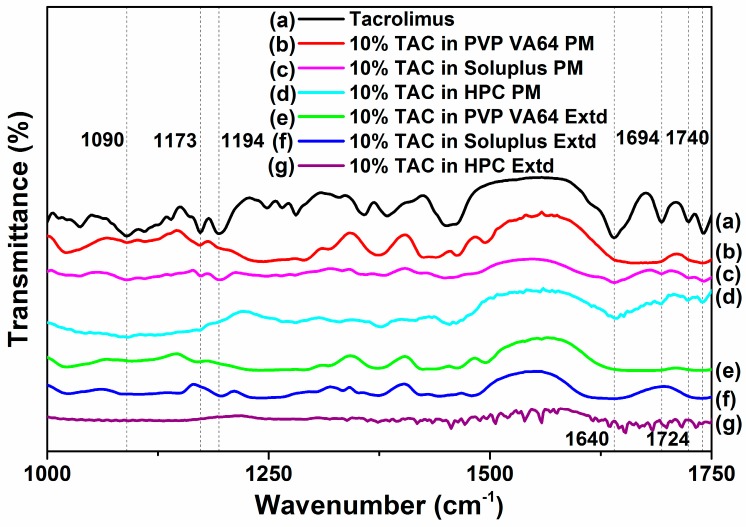
FTIR spectra of TAC and solid dispersion formulations in PVP VA64, Soluplus and HPC.

In the extruded PVP VA64 solid dispersions, the absorption bands attributed to C=O groups at 1724 and 1694 cm^−1^ disappear, and the absorption band at 1640 cm^−1^ was shifted to 1625 cm^−1^. The peaks at 1173 and 1090 cm^−1^ are found in the physical mixture but the peak at 1090 cm^−1^ disappears and the peak at 1173 cm^−1^ is shifted to 1165 cm^−1^ in the extruded solid dispersion. In the extruded Soluplus solid dispersions, the absorption bands attributed to C=O groups at 1724 and 1694 cm^−1^ are present in the physical mixture but disappear in the extruded solid dispersion, and the absorption band at 1640 cm^−1^ was broadened in the extruded sample. The peak at 1090 cm^−1^ is shifted to 1080 cm^−1^ after extrusion while the peak at 1173 cm^−1^ disappears. The peak at 1194 cm^−1^ is slightly shifted to 1197 cm^−1^ in the extruded solid dispersion of TAC in Soluplus. The drug peaks at 1649, 1724 and 1740 cm^−1^ corresponding to the carbonyl groups are present in the HPC physical mixture while in extruded samples the peaks were shifted (Figure 6).

These observations could be attributed to hydrogen bond formation between PVP VA64, Soluplus and HPC with functional groups of TAC especially carbonyl group and hydroxyl group at molecular level [4]. The reduction in peak intensities could also be attributed to the formation of amorphous TAC in the extruded solid dispersions [34].

### 3.7. Dissolution Studies

**Figure 7:**
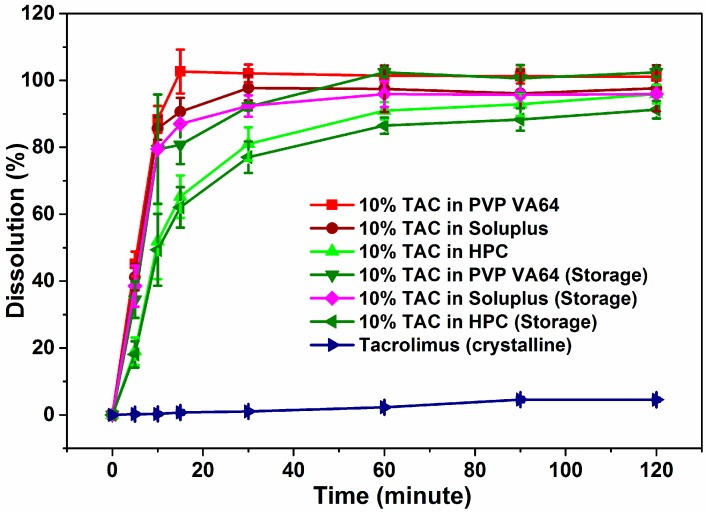
Dissolution profiles of solid dispersions of TAC before and after storage.

The dissolution profiles of crystalline TAC and extruded formulations are shown in Figure 7. Since TAC is practically insoluble in water, 0.1% SLS was added to the dissolution media. About 5% of crystalline TAC had dissolved in media after 2 h of testing while the solid dispersions showed typical burst release of TAC. PVP-VA64, Soluplus and HPC solid dispersions showed 100%, 90% and 65% drug release in the first 15 min. The Soluplus formulation released 98% of drug in the 2 h of testing while the HPC formulation released 96% in the same time. The amorphous TAC in solid dispersion reaches concentrations of 20 mg/L while the crystalline drug reaches only 2 mg/L after two hours of dissolution testing. The HPC solid dispersion showed slowest release followed by Soluplus and PVP VA64 in first 15 min of dissolution testing. This could be due to the slow solublization behavior of the HPC which first swells and then solubilizes in water.

### 3.8. Stability Studies

**Figure 8:**
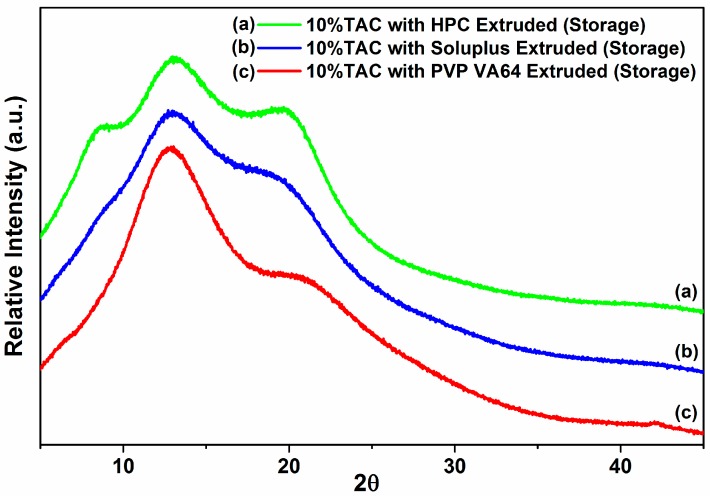
X-ray diffraction patterns of solid dispersion stored at 40 °C and 75% RH for 3 months.

The TAC solid dispersions were stored in screw capped glass bottles in a stability chamber at 40 °C/75% RH and their physical and chemical stability was studied. The % drug content was found to be at 94%, 86% and 82% for TAC solid dispersions in PVP VA64, Soluplus and HPC respectively after 3 months. The X-ray diffractogram showed an amorphous halo for all three solid dispersions after storage at 40 °C and 75% RH (accelerated conditions) for 3 months (Figure 8). This showed that amorphous TAC in all three formulations has good chemical and physical stability on storage under stressed conditions. The T_g_ of TAC was found to be 78.8 °C and calculated T_g_ of PVP-VA 64 solid dispersion was found to be 98.6 °C. As per general convention, storing 50 °C below T_g_ could significantly reduce the molecular mobility and re-crystallization [35]. The solid dispersions exhibited a typical burst release of TAC similar to the initial dissolution profile with approximately 81%, 87% and 62% of drug release in 15 min for PVP VA64, Soluplus and HPC solid dispersions of TAC respectively (Figure 7).

### 3.9. ODT Formulation and Characterization

Two ODT compositions considered for being compressed into tablets are shown in Table 2. These formulations were punched into 250 mg tablets and the hardness, friability and disintegration were tested (Table 3). The disintegration time in all formulations was influenced by the polymer in the following order.

Soluplus < HPC < PVP VA64

It was found that PVP VA64 produced the hardest tablets for each formulation with the least friability and longest disintegration time. It was also observed that increasing the PVP VA64 concentration also contributed to a corresponding increase in the disintegration time (data not shown). The USP stipulates that an ODT should disintegrate within 30 s [36] while the European Pharmacopoeia gives a time limit of 3 min (180 s) [37].

Formulation 2 that showed optimal performance in both hardness and disintegration in all three polymers was chosen and tablets containing solid dispersions of TAC were prepared.

### 3.10. Dissolution Testing of TAC ODTs

**Figure 9:**
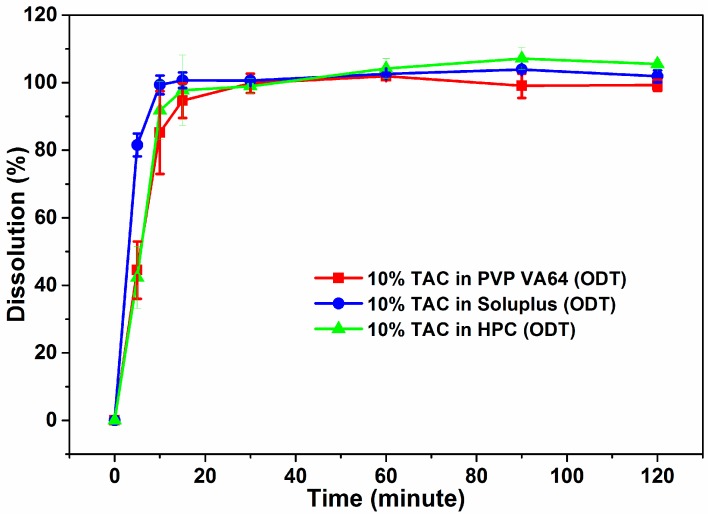
Dissolution profiles of ODTs containing solid dispersions of TAC.

The ODTs were compressed with the polymer component of the blank tablets replaced by the extruded solid dispersion and formulated such that they contained 2.5 mg per 250 mg ODT. All the ODTs showed 100% drug release in the first 30 min of dissolution testing (Figure 9). Slight improvement was observed in rate of TAC release from Soluplus and HPC formulations as compared to powdered solid dispersion where HPC showed 65% release of TAC.

## 4. Discussion

TAC is an established immunosuppressant that is used to prevent acute rejection of transplanted organ especially in the case of liver and lung transplants [38]. It is chemically classified as a macrolide lactone. The BCS places it in class II implying that the bioavailability of the drug is limited by its solubility at the site of absorption [39].

The DSC results of TAC show that the drug melts above 130 °C and on rapid cooling it solidifies into its amorphous form with a T_g_ at 78.8 °C. The extrusion was performed at 140 °C with reduced barrel length to decrease the residence time of material in the heated extruder. The material exiting the die was in the form of flexible, transparent, cylindrical rods of melt which cooled quickly on the conveyor belt to form brittle, glassy strands which were later milled to powders before storage. The clear and transparent visual appearance of the strands could mean that the drug is either dispersed as very small particles in the polymeric melt or has dissolved in the polymeric melt after melting in the extruder. This suggests the possibility of formation of a solid solution where the drug is molecularly dispersed in the polymer [40].

The DSC and XRD results confirm that TAC was present in the amorphous form. The melting peak and characteristic XRD peaks of the drug which were seen in the physical mixtures were not found in the extruded solid dispersions.

The molecular structure of TAC shows hydroxy, carbonyl (ester and ketone) and ether moieties in the molecule. The interaction between the drug and the polymers is observed in the region from 1050 to 1200 cm^−1^ in the FTIR. The changes in the spectra of the extruded solid dispersions suggest possibility of hydrogen bond formation between the carbonyl and hydroxyl groups present in TAC and the polymer. The ester and amide functional groups of PVP VA64 could be involved in hydrogen bonding like interactions during extrusion [41]. These interactions could also play a role in maintaining the super saturation of drug in media on dissolution and inhibition of the recrystallization of drug thereby enhancing stability [14].

The extruded solid dispersions exhibit a higher rate and percentage of dissolution of TAC compared to both the physical mixtures and pure crystalline drug. The solid dispersions showed a burst release of the drug in the first 15 min compared to the slow dissolution of pure crystalline TAC. The dissolution results obtained after the storage of solid dispersions in accelerated conditions for 3 months show dissolution profiles similar to those obtained during the initial testing. The increase in drug dissolution could be attributed to the amorphous form being a higher energy state which has been observed to exhibit higher solubility and dissolution rate compared to its crystalline form and the presence of hydrophilic polymer which enhances the rate of dissolution of drug from the extruded solid dispersions. These amorphous solid dispersions of TAC were incorporated into ODTs. PVP VA64 which acts as a binder in the compressed ODT releases the drug faster when it is tested as a solid dispersion powder and there is a marginally faster rate of drug release from the powdered solid dispersions compared to the ODTs. In the case of Soluplus and HPC, the ODT formulation helps disperse the solid dispersion into the media as opposed to the solid dispersion powder which tends to form lumps in the dissolution tub. This explains the slight improvement in the ODT over the solid dispersion powders in the time taken to release the drug.

## 5. Conclusions

HME was found to be a suitable technique for producing solid dispersions of TAC using PVP VA 64, Soluplus and HPC as carrier polymers at a drug loading of 10% by weight. The solid dispersions were found to be clear, glassy strands of TAC in the carrier polymers where the drug was stabilized in the amorphous form. The extruded solid dispersions which contained TAC in the amorphous form, showed a higher rate of dissolution. These solid dispersions were formulated into ODTs which maintained the dissolution advantage of the solid dispersions and were stable at accelerated conditions for 3 months. This approach can be used to deliver TAC as orally disintegrating tablets with improved dissolution rate.

## Figures and Tables

**Table 1:** Properties of drug and carrier polymers. TAC: Tacrolimus; PVP VA64: Polyvinylpyrrolidone vinyl acetate; HPC: Hydroxypropyl Cellulose.

Drug/Polymer	Aqueous Solubility	Molecular Weight	Glass Transition (°C)	Hansen’s Solubility Parameters (MPa^1/2^)	Interaction Parameter (Δ*δt*)	Solid State
TAC	Insoluble (2.6 µg/mL)	804.02 g/mol	78.8 (amorphous form)	19.1 *	-	Crystalline
PVP VA64	Very soluble	45–70 kD	101	19.7 [31]	0.6	Amorphous
Soluplus	Very soluble	118 kD	70	19.4 [31]	0.3	Amorphous
HPC	Soluble	95 kD	105	21.27 [32]	2.17	Semi-crystalline

* Calculated.

**Table 2:** Composition of orally-disintegrating tablets (ODT) formulations.

Excipient	Formulation 1	Formulation 2
Microcrystalline cellulose	69.75%	59.5%
Crospovidone	10%	10%
Mannitol	10%	20%
Magnesium Stearate	0.25%	0.5%
Polymer/TAC Solid dispersion	10%	10%

**Table 3:** Characterization of blank ODTs.

Formulation	Polymer	Hardness (kP)	Friability (%)	Disintegration Time (s)
Formulation 1	PVP VA64	18.6 ± 2.8	0%	83
	Soluplus	11.9 ± 2.5	0.07%	10
	HPC	20.9 ± 2.7	0.06%	50
Formulation 2	PVP VA64	23.0 ± 1.8	0%	60
	Soluplus	17.5 ± 0.8	0.03%	18
	HPC	17.5 ± 1.0	0%	40
